# Birthing parent adverse childhood experiences and risk of atopic diseases in 5-year-old children

**DOI:** 10.3389/falgy.2024.1483911

**Published:** 2025-01-07

**Authors:** Makayla Freeman, Anna L. MacKinnon, Mark Anselmo, Suzanne Tough, Lianne Tomfohr-Madsen

**Affiliations:** ^1^ Department of Psychology, University of Calgary, Calgary, AB, Canada; ^2^ Department of Educational and Counselling Psychology and Special Education, University of British Columbia, Vancouver, BC, Canada; ^3^ Department of Psychiatry and Addiction, University of Montréal, Montréal, QC, Canada; ^4^ CHU Sainte-Justine Research Center, Montréal, QC, Canada; ^5^ Alberta Children’s Hospital Research Institute, Calgary, AB, Canada; ^6^ Department of Paediatrics, Cumming School of Medicine, University of Calgary, Calgary, AB, Canada; ^7^ Community Health Sciences, Cumming School of Medicine, University of Calgary, Calgary, AB, Canada

**Keywords:** adverse childhood experiences, atopic diseases, intergenerational health outcomes, pregnancy, asthma, allergy

## Abstract

Following up on previous findings from the All Our Families (AOF) cohort, the current study investigated the relationship between birthing parent history of adverse childhood experiences (ACEs) and child atopy, including asthma, allergy, and eczema, at five years of age. Potential indirect effects were explored. Participants completed the ACEs scale, validated questionnaires of anxiety and depression symptoms, and reported on their and their children's atopic disease history. Archival analyses of AOF data (*N* = 3,387) was conducted using logistic regression and path analysis with counterfactually based indirect effects. Birthing parent history of ACEs was associated with an 18% increased risk of child allergy at five years (OR = 1.18, 95% CI: 1.09, 1.20). Exploratory path analyses indicated a significant indirect effect of ACEs through birthing parent history of atopy on child asthma, allergy, and eczema at five years. There were no significant indirect effects through birthing parent symptoms of anxiety or depression during pregnancy, at two or five years postpartum. Birthing parent history of ACEs, combined with birthing parent history of atopy, may elevate the risk of child atopy. This presents an opportunity for early intervention for children at risk of atopic disease.

## Introduction

Atopic diseases, including asthma, allergy, and eczema are the most commonly reported chronic conditions in childhood, with prevalence estimates ranging from 5%–25% between 0 and 10 years old, depending on disease type (1–5). Unfortunately, atopic diseases in childhood present significant health concerns that impact quality of life and increase risk for other poor health outcomes and chronic diseases [e.g., anxiety; (6–10)]. Improving understanding of the complex interplay of processes influencing the development of atopic diseases in childhood could improve prevention, detection, and early intervention efforts. There are some known genetic, obstetric, and environmental risk factors for child atopic disease, including family history, preterm birth, and exposure to irritants/allergens. There is also emerging evidence demonstrating an intergenerational impact of birthing parents' adverse childhood experiences on their children's risk for developing atopic disease (11–14).

Adverse childhood experiences (ACEs) refer to events that may be highly distressing or traumatic (e.g., abuse, neglect, exposure to domestic violence and significant household dysfunction) occurring prior to 18 years of age. ACEs are associated with a range of negative physical (e.g., cardiovascular disease, obesity, asthma) and mental (e.g., mood and anxiety disorders) health outcomes in adulthood (15–21). Research suggests that the risk conferred by ACEs may extend across generations, leading to increased risk for birth complications, developmental delays, as well as internalizing and externalizing problems (22–25). Data from the All Our Families prospective cohort study demonstrated an association between birthing parents' history of experiencing childhood abuse and diagnoses of asthma and allergies in children at the age of 2, as well as indirect effects through perinatal depression and postpartum anxiety (14, 26, 27). More longitudinal evidence is required to clarify the relationship between birthing parent ACEs with the onset and progression of child atopic disease.

Several explanations have been proposed for the intergenerational transmission of the risks conferred by birthing parent history of ACEs. Potential pathways include both genetic factors (e.g., birthing parent history of atopic disease) and environmental factors (e.g., birthing parent depression and anxiety) during the prenatal and early developmental periods, which have been linked with increased risk of child atopic disease (28, 39). Pregnancy represents a sensitive period in which fetal immune system development may be impacted by alterations to the birthing parent neuroendocrine system, nervous system reactivity, and/or Hypothalamic Pituitary Adrenal Axis (HPA) function resulting from ACEs, in addition to genetic inheritance (28–37, 39–43). Additionally, immune system development continues into the postpartum period in which environmental factors, such as birthing parent mental health, impact risk of atopic disease (13, 14, 44–46). Thus, *in utero* programming and early environmental exposures impact susceptibility to the development of atopic disease.

**Figure 1:**
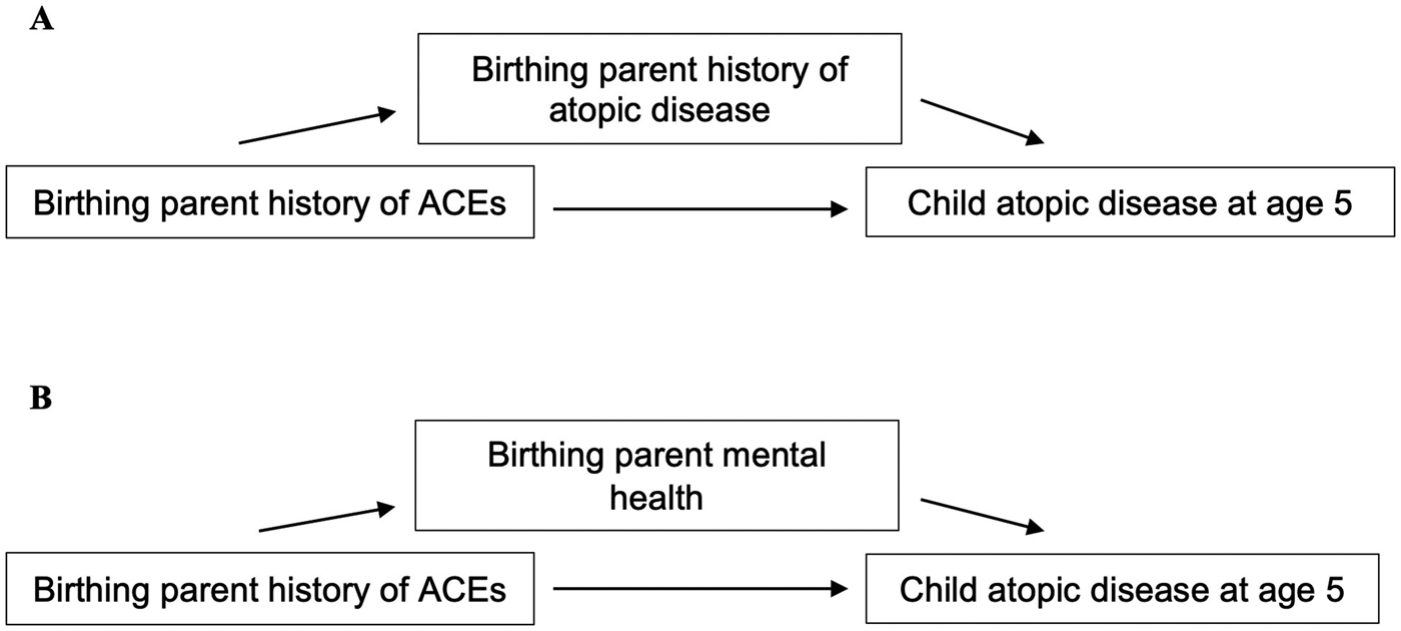
Model of relationship between birthing parent ACEs and child atopic disease at 5 years. **(A)** Model of indirect effects through birthing parent history of atopic disease. **(B)** Model of indirect effects through birthing parent mental health, including symptoms of depression and anxiety.

The current study aimed to investigate whether the relationship between birthing parent ACEs and child atopic diseases persisted when children were five years old, given more definitive diagnoses are possible at this age (47, 48). Based on previous findings, birthing parent ACEs were expected to be associated with an increased risk of diagnoses of child atopic diseases, including asthma, allergy, and eczema, at five years. Potential indirect effects through birthing parent history of atopy as well as birthing parent mental health, including symptoms of depression and anxiety, were also explored (see Figure 1).

## Method

### Participants and procedures

The current investigation utilizes longitudinal data from the All Our Families (AOF) cohort study and was approved by the Conjoint Health Research Ethics Board (CHREB) at the University of Calgary (REB19-1646) (26, 27). Given the All Our Families (previously All Our Babies) study was designed to be an epidemiological prospective cohort study, the largest sample possible was recruited from all “women” accessing prenatal care in Calgary, Canada between 2008 and 2011, to enable longitudinal analyses (26). A total of 4,011 individuals responded to community advertisements or researchers at primary health care offices and laboratory services recruiting “pregnant women” (hereafter referred to as birthing parents, as gender identity information was not collected), of which 3,387 met inclusion criteria and were enrolled in the study (26). Eligibility criteria included being less than 25 weeks gestation, at least 18 years of age, able to complete questionnaires in English, and receiving prenatal care in Calgary, Alberta. Participants completed a battery of questionnaires before 25 weeks gestation (early pregnancy), at 34–36 weeks gestation (late pregnancy), at 4 and 12 months postpartum, and at 2, 3, and 5 years postpartum.

### Measures

#### Demographics

Relevant sociodemographic information was collected via self-report in early pregnancy, including birthing parent ethnicity (coded as European-Canadian or not), education (coded as ≥ post-secondary), household income (coded as ≥$80,000 CAD) (14, 49), marital status (coded as partnered or not), and parity (coded as primiparous or not). At four months postpartum, participants reported on gestational age at birth (preterm <37 weeks) and infant sex (coded as male or not). Participants reported on breastfeeding duration (weeks) at 12 months postpartum.

#### Birthing parent history of adverse childhood experiences

At 3 years postpartum, the Adverse Childhood Experiences (ACE) scale was administered. The ACE scale is an 11-item retrospective self-report questionnaire that measures eight categories of child abuse and household dysfunction before the age of 18 (15, 50). Some items (e.g., “Were your parents separated or divorced?”) are rated dichotomously (0 = no, 1 = yes) and others (e.g., “How often did anyone at least 5 years older than you or an adult ever touch you sexually?”) are rated on a 3-point frequency scale (1 = never, 2 = once, 3 = more than once). Participants were coded as having experienced zero to four or more categories of ACEs (continuous variable of 0 to ≥4) in accordance with previous research that notes a dose-response relationship between ACEs and mental health outcomes (15, 50, 51). The ACE questionnaire is widely used and has demonstrated satisfactory consistency and test-retest reliability (52).

#### Birthing parent mental health

Symptoms of anxiety and depression measured using the Spielberger State Anxiety Inventory (STAI) and the Edinburgh Postnatal Depression Scale (EPDS), respectively, during late pregnancy and at 2 and 5 years postpartum were included in the current investigation (53, 54). The EPDS is a 10 item self-report questionnaire used to measure perinatal and postnatal depression. Items are rated on a 4-point scale and summed to produce a total score ranging from 0–30, wherein higher scores indicating more depressive symptoms (53, 55). The STAI is a 20-item self-report measure of state anxiety, with items rated on a 4-point scale (ranging from “Almost Never” to “Almost Always”) based on “how you feel right now” (54). Responses are summed to calculate a total score ranging from 20–80, wherein higher scores indicate higher state anxiety. Both the STAI and the EPDS have demonstrated satisfactory validity and reliability during the perinatal period (53, 56–61). Total continuous scores for the STAI and the EPDS were used for all analyses.

#### Atopic disease

At 5 years postpartum, participants reported on their own history of atopic disease, including asthma and allergy (coded as 0 = none, 1 = either or both). Participants were also asked if their child had experienced asthma, allergies (environmental or food), or eczema (dermatitis/psoriasis) within the past year (between 4 and 5 years old) to ensure the measure did not capture experiences of childhood wheeze (38, 47). Participants responded no or yes (coded as 0 or 1, respectively) to each disease outcome.

### Statistical analysis

Descriptive statistics were conducted using IBM SPSS Statistics [Version 27; (62)]. Differences in demographic and birthing parent characteristics among children with and without atopy at 5 years old were tested for descriptive purposes using independent *t*-tests for continuous variables (birthing parent age, breastfeeding duration, symptoms of anxiety and depression) and *χ*2 tests for dichotomous variables (birthing parent ACEs and history of atopy, birthing parent ethnicity, education, household income, marital status, parity, preterm birth, and infant sex). Logistic regressions and path analyses were conducted with Mplus 8, as this program can handle binary outcomes and mediators (63). Logistic regressions [using the Categorical option to specify the dependent variable; (64)] tested if birthing parent ACEs were associated with risk for atopy (asthma, allergies, and eczema, respectively) in children at 5 years of age. Odds ratios were estimated, where significance is indicated by a 95% confidence interval (CI) that does not include one. Birthing parent history of atopy, ethnicity, education, household income, parity, gestational age, and child sex were included as covariates in adjusted models since they have been associated with risk for child atopic disease (34, 38, 65–72). Exploratory path analyses (using the Model Indirect command (64); tested indirect effects of birthing parents' history of ACEs on their children's risk for atopic disease through birthing parent history of atopy and birthing parent mental health, including anxiety and depression during late pregnancy and at 2 and 5 years postpartum, respectively. Total natural indirect effects (TNIE) were derived using counterfactuals (i.e., the contrast between the effect of the mediator on the outcome at different levels of the exposure), which is the Mplus default for logistic regression (72). Models were estimated using 1,000 bootstrapped resamples, where significance is indicated by a 95% confidence interval (CI) that does not cross zero (74). Missing data was handled using full information maximum likelihood (FIML), which produces unbiased model parameters (75).

## Results

### Sample description

The AOF cohort is representative of the pregnant population in an urban centre in (1, 27, 76). Of the 3,387 participants who reported demographics in early pregnancy, most (81%) completed post-secondary schooling, most (78.6%) identified as European-Canadian (with 4.4% identifying as Chinese, 3.5% Mixed/Other, 3.0% South Asian, 2.3% Latin American, 1.9% Filipino, 1.5% Southeast Asian, 1.5% Black/African North American, 1.3% Arab, 0.9% First Nations/Metis, 0.1% Korean, 0.4% West Asian, and 0.3% Japanese), almost all (98.6%) reported having a partner, and half (50.0%) were pregnant with their first child.

Of the participants who reported on history of ACEs (*n* = 1,984), 62.3% (*n* = 1,237) reported experiencing at least one ACE and 14.8% (*n* = 294) reported four or more ACEs. Of the participants who reported on children's atopic disease at 5 years (*n* = 1,960), 31.4% (*n* = 616) reported having a child with an atopic disease: 7.3% (*n* = 144) with asthma, 9.4% (*n* = 319) with allergies, and 18.3% (361) with eczema, respectively. Birthing parents of children with asthma reported less education, were less likely to identify as European-Canadian, and were more likely to report one or more ACEs. Birthing parents of children with allergies reported more ACEs. Birthing parents of children with asthma, allergy, or eczema were also more likely to report a history of asthma or allergy themselves. Characteristics of the sample according to child atopic disease are presented in Table 1.

**Table 1:** Characteristics of the sample according to child atopic disease at 5 years old.

Variable	Asthma		Allergy		Eczema	
	M (SD) or *n* (%)		M (SD) or *n* (%)		M (SD) or *n* (%)	
	No (*n* = 1,818)	Yes (*n* = 144)	No (*n* = 1,647)	Yes (*n* = 319)	No (*n* = 1,607)	Yes (*n* = 361)
Adverse Childhood Experiences						
No Reported ACEs^a^	594 (38.6)	**35** (28.7)*	548 (39.3)	**83** (31.1)*	517 (37.9)	116 (38.4)
Any Reported ACEs^a^	945 (61.4)	**87** (71.3)*	848 (60.7)	**184** (68.9)*	846 (62.1)	186 (61.6)
4 or More Reported ACEs^b^	211 (13.7)	18 (14.8)	179 (12.8)	**49** (18.4)*	184 (13.5)	44 (14.6)
Demographic & health information						
Birthing parent age (years)*	30.85 (4.34)	31.06 (4.54)	30.88 (4.33)	30.83 (4.51)	30.95 (4.38)	30.50 (4.24)
Ethnicity (European-Canadian)	1,494 (82.5)	**107** (75.4)*	1,348 (82.2)	255 (80.4)	1,319 (82.5)	286 (79.7)
Education (≥post-secondary)	1,456 (80.5)	**100** (70.4)**	1,309 (80.0)	251 (79.2)	1,278 (80.0)	285 (79.4)
Household income (≥$80,000)	1,284 (73.5)	100 (73.0)	1,162 (73.3)	224 (73.9)	1,129 (73.2)	259 (74.2)
Marital status (partnered)	1,794 (99.3)	138 (97.9)	1,623 (99.1)	313 (99.3)	1,585 (99.2)	354 (99.4)
Parity (primiparous)	897 (49.8)	**84** (60.0)*	805 (49.3)	**180** (57.5)**	800 (50.3)	187 (52.5)
Preterm birth	110 (6.3)	**16** (11.4)*	108 (6.8)	18 (5.7)	105 (6.8)	21 (6.0)
Infant sex (male)	947 (52.1)	80 **(**55.6)	851 (51.7)	178 (55.8)	838 (52.2)	192 (53.2)
Breastfeeding (weeks)*	29.89 (15.67)	**24.98** (15.47)*	29.71 (15.61)	28.34 (16.10)	30.11 (15.70)	**26.92** (15.45)*
Birthing parent history of atopy	444 (24.9)	**71** (51.8)***	381 (23.6)	**129** (41.6)***	383 (24.4)	**127** (35.8)**
Birthing Parent Mental Health*						
Depression symptoms in pregnancy	4.75 (4.13)	5.01 (4.48)	4.70 (4.18)	5.14 (4.01)	4.75 (4.17)	4.82 (4.07)
Anxiety symptoms in pregnancy	31.78 (8.81)	32.01 (8.72)	31.76 (8.88)	32.00 (8.37)	31.78 (8.92)	31.83 (8.18)
Depression symptoms at 2 years	7.49 (6.90)	8.09 (6.98)	7.46 (6.88)	8.00 (7.02)	7.45 (6.81)	7.91 (7.27)
Anxiety symptoms at 2 years	30.30 (8.38)	31.32 (8.09)	30.32 (8.46)	30.75 (7.91)	30.49 (8.50)	29.91 (7.86)
Depression symptoms at 5 years	8.49 (8.31)	8.73 (8.58)	8.43 (8.32)	9.04 (8.44)	8.45 (8.25)	8.83 (8.69)
Anxiety symptoms at 5 years	9.34 (3.31)	9.42 (3.42)	9.30 (3.31)	9.63 (3.35)	9.35 (3.33)	9.34 (3.26)

*Note*. M (SD) = mean (standard deviation)*.

^a^ indicates that *χ*2 tests were conducted between participants who reported any ACEs and those who reported none.

^b^ indicates that *t*-tests were conducted between participants who reported 4 or more ACEs and those who reported 3 or less ACEs. Statistical significance is indicted by boldface.

* *p* < .05.

** *p* < .01.

*** *p* < .001.

### Logistic regression

Birthing parent ACEs were not associated with child asthma in either unadjusted (OR = 1.10, 95% CI: .97, 1.23) or adjusted models (OR = 1.03, 95% CI: .91, 1.18). Similarly, birthing parent history of ACEs was not significantly associated with child eczema in unadjusted (OR = 1.02, 95% CI: .93, 1.11) or adjusted models (OR = 1.01, 95% CI: .92, 1.10). However, birthing parent ACEs were significantly associated with child allergies at 5 years old in both unadjusted (OR = 1.18, 95% CI: 1.09, 1.30) and adjusted models (OR = 1.18, 95% CI: 1.06, 1.29). See Table 2 for complete presentation of the binary logistic regression results.

**Table 2:** Logistic regression models of birthing parent ACEs history on child atopy at 5 years.

	Asthma	Allergy	Eczema
	OR [95% CI]	OR [95% CI]	OR [95% CI]
Unadjusted			
Birthing parent history of ACEs	1.10 [.97, 1.23]	**1.18 [1.09, 1.30]**	1.02 [.93, 1.11]
Adjusted			
Birthing parent history of ACEs	1.03 [.91, 1.18]	**1.18 [1.06, 1.29]**	1.01 [.93, 1.11]
Birthing parent history of atopy	**2.07 [1.62, 2.56]**	**1.65 [1.36, 1.97]**	**1.49 [1.25, 1.79]**
Ethnicity (European-Canadian)	.64 [.43, 1.02]	.85 [.62, 1.19]	.80 [.60, 1.10]
Education (≥post-secondary)	**.57 [.37, .88]**	1.06 [.75, 1.48]	.96 [.73, 1.30]
Household income (≥$80,000)	1.16 [.77, 1.85]	1.07 [.78, 1.44]	1.07 [.81, 1.41]
Parity (primiparous)	**.65 [.45, .92]**	**.73 [.57, .95]**	.94 [.72, 1.18]
Gestational age (weeks)	**.91 [.85, .99]**	1.02 [.95, 1.11]	1.05 [.98, 1.13]
Child sex (male)	1.18 [.85, 1.68]	1.22 [.96, 1.60]	1.06 [.84, 1.32]

*Note*. Odds ratios with confidence intervals that do not cross 1 are significant. Bolded estimates indicate significance.

OR, odds ratio; CI, confidence internal.

### Exploratory path analysis

Indirect effects are presented in Table 3. After adjusting for birthing parent ethnicity and education, household income, parity, gestational age, and child sex, there were significant indirect effects of birthing parents' ACEs through birthing parent history of atopy on increased risk of children's asthma, allergies, and eczema at 5 years old. There were no significant indirect effects through birthing parent mental health during pregnancy, at 2 or 5 years postpartum for children's asthma, allergies, or eczema.

**Table 3:** Counterfactually based indirect effects of birthing parent atopy and mental health.

	Asthma		Allergy		Eczema	
	TNIE [95% CI]	OR [95% CI]	TNIE [95% CI]	OR [95% CI]	TNIE [95% CI]	OR [95% CI]
Birthing parent history of atopy	**.002 [.000, .005]**	**1.02 [1.004, 1.042]**	**.002 [.001, .004]**	**1.01 [1.003, 1.027]**	**.002 [.000, .004]**	**1.01 [1.002, 1.022]**
Anxiety symptoms in pregnancy	.000 [−.002, .02]	.99 [.98, 1.02]	.000 [−.002, .002]	1.00 [.97, 1.02]	.000 [−.002, .002]	1.001 [.99, 1.02]
Depression symptoms in pregnancy	.000 [−.002, .003]	1.002 [.98, 1.03]	.001 [−.001, .003]	1.01 [.99, 1.03]	.000 [−.002, .003]	1.001 [.99, 1.02]
Anxiety symptoms at 2 years	.001 [.000, .004]	1.01[.99, 1.03]	.000 [−.001, .002]	1.00 [.99, 1.02]	−.001 [−.003, .001]	.99 [.98, 1.01]
Depression symptoms at 2 years	.001 [−.001, .003]	1.01 [.99, 1.03]	.001 [−.001, .003]	1.01 [.99, 1.03]	.001 [−.001, .004]	1.01 [.99, 1.03]
Anxiety symptoms at 5 years	.000 [−.002, .001]	1.00 [.99, 1.01]	.001 [.000, .002]	1.01 [.99, 1.02]	.000 [−.002, .001]	1.00 [.99, 1.01]
Depression symptoms at 5 years	.000 [−.003, .002]	.99 [.97, 1.02]	.000 [−.001, .002]	1.00 [.99, 1.02]	.001 [ −.002, .003]	1.01 [.99, 1.02]

*Note*. Each row represents a separate model. Indirect effects with confidence intervals that do not cross 0 are significant, whereas odds ratios with confidence intervals that do not cross 1 are significant. Bolded estimates indicate significance. All models included birthing parent ethnicity, education, household income, parity, gestational age, and child sex as covariates.

TNIE, total natural indirect effect; OR, odds ratio; CI, confidence internal.

## Discussion

This follow-up study examined the relationship between birthing parents' ACEs and their children's risk of atopic disease at 5 years old, in the All Our Families cohort, and explored potential genetic and environmental pathways of intergenerational transmission. Results indicated that birthing parent ACEs were directly associated with an increased risk of allergies at 5 years, but not asthma or eczema, beyond other known correlates of atopic disease in children. There was an indirect effect of ACEs on children's asthma, allergy, and eczema at 5 years, with indirect effects observed through birthing parent history of atopy, but not symptoms of birthing parent mental health.

The current findings indicated that the association between birthing parent history of childhood abuse and child asthma at 2 years of age, observed in a previous report with this cohort (14), did not persist at five years. However, asthma diagnoses at two years could have been inflated due to the prevalence of childhood wheeze (36, 38). There is a reduced probability of childhood wheeze being captured by the measure of atopic disease in the current study, as a more definitive diagnosis of asthma is possible at 5 years of age (47, 48). Birthing parent ACEs was also not directly associated with child eczema at 5 years. However, the relationship may be better explained by indirect effects, such as birthing parent history of atopy as examined in the exploratory path analyses. It is also possible that other risk factors not included in the current study, such as environmental exposures (e.g., climate, pollution, microbial exposure), allergen exposure (e.g., animal dander), and family history of atopic disease (beyond the birthing parent), play role a bigger role as children age and should be investigated (3, 5, 77, 78). Birthing parent history of ACEs was directly associated with birthing parents' reports of child allergies at 5 years, which corresponds with the previous findings from this cohort (14), and the observed increases in effect size were aligned with what has been previously published in studies of child atopic disease outcomes (12, 19, 21). It is possible that the measurement of birthing parent childhood abuse in the previous study and ACEs in the current investigation are differentially related to child atopic diseases; the ACEs questionnaire was administered at 3 years postpartum, as compared to the childhood abuse questionnaire administered during pregnancy, and captures experiences of neglect and household dysfunction in addition to childhood abuse (79).

The exploratory path analyses suggest that birthing parent history of atopy may represent a genetic pathway through which birthing parent exposure to ACEs confers risk for children's development of atopic disease. These findings align with research that demonstrates that ACEs are associated with an increased risk of atopic disease onset and that family history of atopic disease is a risk factor for the development of child atopic disease (19, 28, 29, 31, 38, 80). Due to the high heritability of atopic disease (5, 29, 38), it is possible that a genetic predisposition to atopy is animated by exposure to ACEs, which then increases intergenerational risk of transmission through pathways such as disrupted maternal cortisol production and/or immune disruption during pregnancy, which impact fetal immune-system development (30, 32). It is also possible that greater exposure to ACEs increases the likelihood of individuals developing atopy, which in turn increases the risk that their children will develop asthma, allergies, or eczema by 5 years of age (19, 28, 29, 31, 38, 80). As the relationship between genetic and environmental factors in the development of atopic disease is bidirectional, future studies should conduct cross-lagged panel analyses to disentangle the longitudinal direction of the relationship between birthing parent atopic disease and exposure to ACEs.

Despite prior findings that birthing parent mental health was significantly associated with risk of child atopic disease at 2 years of age (14), we found no significant associations or indirect effects through birthing parent mental health and child atopic disease at 5 years old. It may be that birthing parent mental health has less influence on children's atopy at 5 years than other environmental factors not included in the current study, such as airborne pollutants (77, 81–83). Other parental factors, such as parenting, may exert greater influence at age 5 and interact with environmental factors not included in the current study to impact child risk of atopy (84, 85). In addition, the current sample has been found to have relatively high and stable levels of social support, which may impact risk for symptoms of depression and anxiety (79, 86, 87). Future investigations should consider clinical samples to ensure that findings are generalizable to subpopulations with higher rates of depression and anxiety (27, 76). Additionally, future studies should examine the interactions between parental factors, such as mental health and parenting, and environmental factors to better understand the joint contributions of social and physical environmental characteristics to child risk of atopic disease.

The findings of the current study have important implications for paediatric and family health care. Having a better understanding of birthing parents' psychosocial and medical history may help to identify children at higher risk of developing atopic diseases and provide an opportunity for early intervention. While there are multiple factors that impact risk for child atopic disease not included in this study, the current investigation suggests that birthing parent history of ACEs and atopic disease are important factors. Identifying children at higher risk of atopic disease may be useful for future research on prevention strategies and guidelines, as targeting high risk populations presents benefits to clinical trials (i.e., smaller sample size, participants motivated to adhere to intervention (88, 89). Providing information on the prevention and management of atopic disease to birthing parents with a history of ACEs, asthma, or allergy could decrease pediatric exposure to known risk factors for child atopy and mitigate intergenerational transmission of risk (36, 85, 90–92). Other findings from the AOF cohort suggest that interventions aimed to build social support may further reduce the impact of birthing parent ACEs on infant health outcomes (85). Lastly, ensuring trauma-informed care is available to patients with a history of ACEs is a critical part of reducing the inequities that may result from ACEs exposure and its sequelae (93–95). The current findings highlight an opportunity to reduce intergenerational health inequities resulting from childhood adversity by providing early intervention to children at higher risk of developing atopic diseases.

### Strengths and limitations

The large sample size and prospective design of the All Our Families cohort study enabled a robust test of the unique impact of birthing parent ACEs on child atopy, beyond several known risk factors, as well as exploration of potential indirect effects using an advanced statistical approach. However, the findings from the current investigation should be interpreted with consideration of some limitations. Participants only reported on atopic diseases that children had experienced between the ages of four and five years old. It is possible that parents of children with persistent atopic disease that had presented before the age of four may not have reported the diagnosis due to the phrasing of the measure, resulting in an underestimation of the rates of child atopic disease in our sample. While parent-report of child atopic disease is commonly used in epidemiological research and has been found to be a valid measure (96–98), physician-reported diagnosis would provide a more reliable measure of atopic disease diagnosis. While the rates of child atopic disease in our sample was representative of national prevalence rates (1–5), future studies should consider including data obtained from health records and ensure that measures include all current and active diagnoses of child atopic disease at the time of data collection. The AOF sample is representative of a pregnant urban population of parents in Canada with access to public health care and results may not be generalized to rural populations or those with limited health care access (14, 35). Future studies should ensure representation from socioeconomically and ethnically diverse families to address inequities in health care access and other social determinants of health. Lastly, other important factors that are known correlates of child atopic disease, including parenting, children's ACEs, and environmental factors such as allergen exposure, airborne pollutants, diet, climate-related factors, which were not included in the current study should be considered in future studies to offer a more holistic view of the various interacting contributors to disease risk (19, 77, 81, 85, 99, 100). Further research, addressing these limitations, will enable a more nuanced and complete understanding of the relationship between parent ACEs and child atopic disease.

## Conclusions

Birthing parents' own exposure to adverse childhood experiences may elevate the risk of their children developing allergy at 5 years. Birthing parent's exposure to adverse childhood experiences may also elevate the risk of their children developing atopic disease, including asthma, allergy, and eczema, through their own history of atopy. Identification of birthing parent history of ACEs and atopic disease during the perinatal period presents an opportunity for early intervention among children at risk of developing atopic disease.

## Data Availability

The datasets presented in this study can be found in online repositories. The names of the repository/repositories and accession number(s) can be found below: https://www.maelstrom-research.org/study/aof.
